# A survey of plants and plant products traditionally used in livestock health management in Buuri district, Meru County, Kenya

**DOI:** 10.1186/1746-4269-8-39

**Published:** 2012-10-08

**Authors:** Martin Muthee Gakuubi, Wycliffe Wanzala

**Affiliations:** 1 Department of Natural Sciences, Faculty of Science, The Catholic University of Eastern Africa, P.O. Box 62157–00200, Nairobi, Kenya; 2 Department of Biological Sciences, School of Pure and Applied Sciences, South Eastern University College (A Constituent College of the University of Nairobi), P.O. Box 170–90200, Kitui, Kenya

**Keywords:** Traditional animal healthcare, Livestock industry, Animal diseases, Plants and plant products, Meru people

## Abstract

**Background:**

Up till now, nomadic communities in Africa have been the primary focus of ethnoveterinary research. Although mainly arable and/or mixed arable/pastoral farmers, Ameru of central Kenya are known to have a rich history of ethnoveterinary knowledge. Their collective and accumulative ethnoveterinary knowledge (EVK) is likely to be just as rich and worth documenting. The aim of the study was to document and analyse the ethnoveterinary knowledge of the Ameru.

**Methods:**

Non-alienating, dialogic, participatory action research (PAR) and participatory rural appraisal (PRA) approaches involving 21 women and men aged between 50 and 79 years old were utilized. A combination of snowball and purposive sampling methods were used to select 21 key respondents. The methods comprised a set of triangulation approach needed in EVK for non-experimental validation of ethnoknowledge of the Ameru.

**Results:**

A total of 48 plant species distributed in 26 families were documented with details of diseases/ill-health conditions, parts of plants used and form of preparation and administration methods applied to different animal groups. Of these families, Fabaceae had the highest number of species (16.67%), followed by Solanaceae (12.5%), Asteraceae and Euphorbiacea (each comprising 8.33%), Lamiaceae (6.25%), Apocynaceae and Boraginaceae (each comprising 4.17%), while the rest of the 19 families, each was represented by a single plant species. About 30 livestock diseases/ill-health conditions were described, each treated by at least one of the 48 plant species. Most prevalent diseases/ill-health conditions included: - anaplasmosis, diarrhea, East Coast fever, pneumonia, helminthiasis, general weakness and skin diseases involving wounds caused by ectoparasites.

**Conclusion:**

The study showed that there was a rich knowledge and ethnopractices for traditional animal healthcare amongst the Ameru. This study therefore provides some groundwork for elucidating the efficacy of some of these plants, plant products and ethnopractices in managing livestock health as further research may lead to discovery of useful ethnopharmaceutical agents applicable in livestock industry.

## Background

Traditional animal healthcare system (also known as ethnoveterinary medicine (EVM)) is as old as the history of domestication of animals [1]. EVM refers to centuries’ old inter-and multidisciplinary components of health that are holistic in application and comprises local ethnoknowledge and associated skills, techniques, practices, beliefs, taboos, cultures, practitioners and socio-economic structures pertaining to the healthcare and healthful husbandry of food-, work- and other income-producing animals [2,3]. EVM has evolved through human civilization processes with a view to improving human well-being via increased benefits from stock raising [3]. Amongst the Meru people, EVM has long existed in various forms and levels [4,5] and transferred from generation to generation by word of mouth, apprenticeship and initiation ceremonies depending on ethnicity [6]. Its documentation and storage is purely based on one’s ability to remember the acquired ethnoveterinary knowledge. This method of archiving, preserving and disseminating such important communal and individual knowledge is however challenging and unsustainable. Rapid technological, environmental, socio-economic, agricultural and cultural changes taking place worldwide, pose further challenges to the future survival and sustainability of EVM [6,7]. For instance, the EVM of the Ameru is faced with a lot of challenges, among them is the expanded range of arable farming activities, which threaten the survival of grasslands, animals, woodlands, microorganisms, bushes and forests, which are the sources of ethnopharmacologically active agents upon which the successful practice of EVM is based [7]. Other factors threatening the survival and sustainability of EVM of the Ameru include: - (1), untimely deaths of resource persons with undocumented ethnoknowledge, (2), extinction of specific plant and animal species and practices for ritual medicines, (3), encroachment of development on and modernization of cultural and traditional life, (4), adoption of lifestyles and education systems that do not embrace ethnoknowledge, (5), shifting bias in religious beliefs, (6), perception of certain socio-cultural practices as unhygienic, witchcraft and satanic and (7), cost- and health-related risks involved in certain socio-cultural ethnopractices. Under these circumstances therefore, there is need to develop stringent documentation and preservation mechanisms of such threatened and yet very useful ethnoknowledge of health [7-9] so that the current generations may not helplessly witness its extinction. For this reason therefore, our study was undertaken to evaluate plant-based ethnoproducts used to manage livestock health by the Ameru of Meru County in central Kenya. It was hypothesized that the findings may provide useful information for further scientific research to determine efficacies for documented ethnoproducts and practices to help improve animal health and human livelihood in Africa.

## Methods

Before the start of this project, prior informed consent was sought from individual key respondents through the local administration in the office of the president, Government of Kenya.

### Description of the study area

#### Meru people and their geographical location

**Figure 1:**
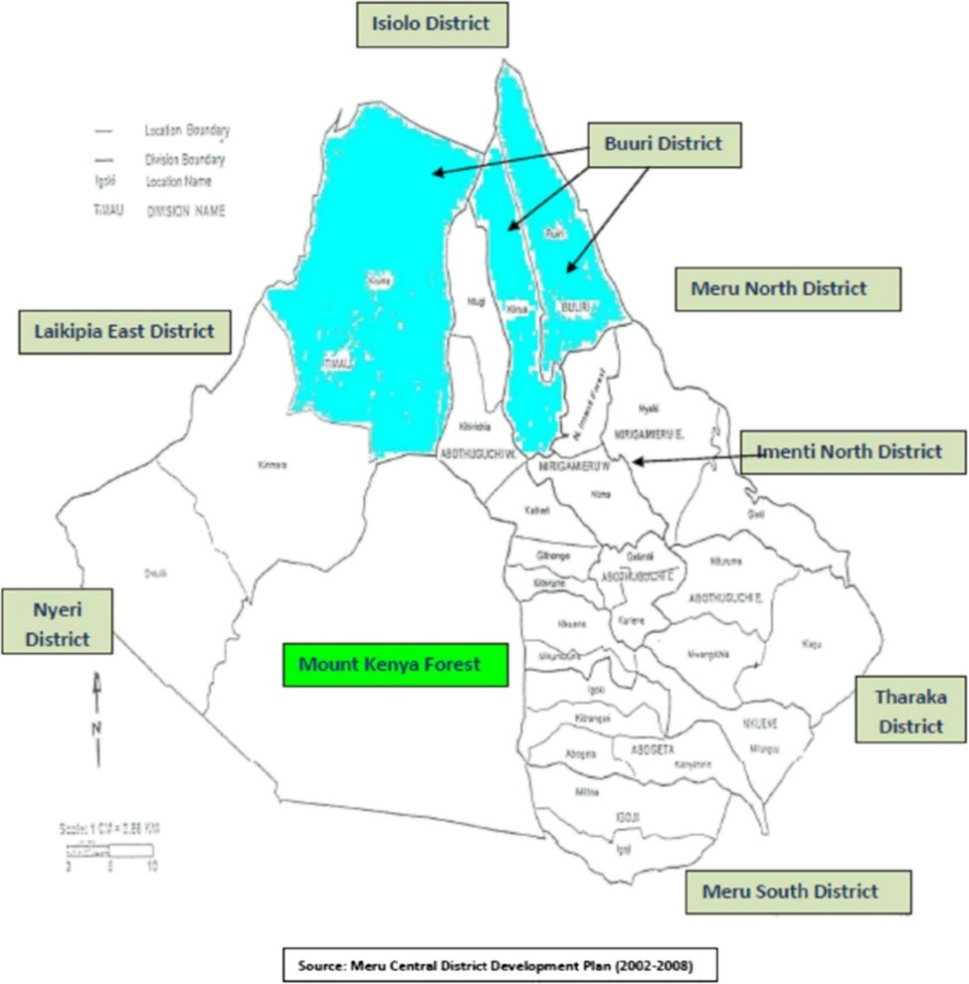
Map of the study area showing the larger Meru region of Kenya, the Buuri district and the neighboring districts.

The name “Meru” refers to both the people and geographical location. The Ameru are part of the Bantu people of East Africa living on the fertile agricultural north and eastern slopes of Mount Kenya within the geographical coordinates of 0° 30' 0" North, 37° 39' 0" East and an altitude of 5,199 m asl. The Meru region constitutes a large area stretching northward to the volcanic Nyambene Hills and southward to the Thuchi River (Figure 1), with the highest point being the summit of Mount Kenya, which greatly influences climate of the area. The rainfall pattern is bimodal with long periods of rain occurring from mid-March to May and short periods occurring from October to December. The mean annual rainfall is about 1,300 mm per year, ranging from 380 mm per year in lowland areas (which includes much of Buuri district that lies on the leeward side of Mount Kenya) to 2,500 mm per year on the north and eastern slopes of Mount Kenya. The climate of Meru region comprises cloudy days with annual temperatures ranging from 10°C around the mountain to 30°C in the lower parts of Meru, relative humidity of 68% and wind of NE at 4 mph.

The Ameru population is about 1.5 million people with a population density ranging from 100 persons per square kilometer in lowland areas to over 400 persons per square kilometer in highland areas. Ameru is a composition of Tigania, Igembe, Imenti, Miutuni, Igoji, Mwimbi, Muthambi, Chuka and Tharaka sub-tribes, which generally speak *Kimîîru* dialects, a Bantu language in the Niger-Congo family [4,5]. The southern dialects of the Ameru are very close to Bantu-speaking Kikuyu people while those of the Northern part show some Cushitic tendencies [4,5]. Although the Chuka and Tharaka sub-tribes have a slightly different oral histories and mythology [4,5], the Imenti sub-tribe dialect dominates in the entire Meru region. The differences in the culture, taboos and language phonetics amongst the sub-tribes of Ameru reflect the varied Bantu origins and influences from the neighbouring Cushite and Nilotic people, as well as different Bantu-speaking neighbours such as the Kikuyu and Embu tribes. Nevertheless, the Meru people exhibit a much older Bantu characteristic phenomenon in grammar and phonetic forms than any other languages of the Bantu-speaking neighbours [10]. Ameru freely combines both arable and pastoral life forms for their socio-economic development.

#### The Buuri district

*Buuri* is a *Kimîîru* word from which the district got its name, which means “dry land”. According to Nyaga [4,5] much of the Buuri district is very dry due to the fact that it lies on the leeward side of Mount Kenya and thus receives very little rainfall (Figure 1). The actual population in Buuri district is not precisely known but the Meru Central District Development Plan (2002–2008) projected the population in 2008 to be roughly 276,000 people.

### Vegetation and soil of Buuri district

Much of Buuri district is dominated by scattered trees, stretches of dry grass and shrubs as the main vegetation types with a number of forests in the neighbourhood, the largest being Mount Kenya forest. The topography of the district was largely influenced by the volcanic activity of Mount Kenya. The dominant soil type is the deep red loam soils, which are well drained and fairly fertile. These vegetation types are the main sources of ethnobotanical products traditionally used in healthcare systems for both humans and animals [5].

### Ethnohealthcare system of Ameru

The traditional healthcare system of the Ameru comprised a wide range of categories of ethnopractitioners such as: - diviners *(kiruria)*, curse detectors *(aringia)* and specialized medicine men *(mugaa)* who were considered integral to the Meru social structure of administration, but the *Mugwe*, the prophet and spiritual leader of each sub-tribe, fulfilled the most important role of both spiritual and physical healing [4,5]. On other hand, *mugaa* was specifically trained in ethnomedicines and healing powers and was widely consulted, particularly for inexplicable illnesses affecting both animals and humans [11].

### Sources of traditional animal healthcare information

Knowledge of ethnoveterinary medicine was surveyed and documented from a varied number of sources in the study area. The identification of sources of information, from which key respondents were selected, included local veterinarians, para-veterinarians and agricultural extension officers responsible for providing extension services to livestock farmers within Buuri district. Meetings of local administration in the office of the President, Government of Kenya were attended and got useful leading information to the identification of potential key respondents. Local livestock traders and dealers, as well as individual livestock farmers, contributed their knowledge of ethnoveterinary medicine based on their professional and economic activities, whereas church leaders, community/village/clan leaders (*Koomenjoe*) and Meru council of elders (*Njuri-Ncheke*), had also very useful leading information on traditional animal healthcare system of the Ameru. Local ethnopractitioners, including general traditional healers/herbalists (*Ndagitari wa miti*), diviners *(Kiruria)*, curse detectors *(Aringia)*, specialized medicine men *(Mugaa),* spiritualists/ritualists (*Nkoma cia bajuju*) and prophet and spiritual leaders (*Mugwe*), formed a particular special subset of knowledgeable people from whom key respondents were also drawn. Secondary data were also considered a very important source of leading information and at Meru County Veterinary Office (CVO), records on traditional animal healthcare system of the Ameru were accessed and utilized. All these groups were consulted because each was associated with a specific aspect of ethnoveterinary knowledge relevant to the study.

### Composition of the 21 key respondents

A survey study was conducted in Buuri district, Meru County during the months of April and May 2011. Ethnopractitioners offering primary healthcare services to local livestock industry were considered the target key respondents in the study and the selection process was based on the knowledge base, experience and current practices in ethnoveterinary medicine of the target individual. The first step in this study was the generation of a purposive sample of the key respondents from a wide range of sources mentioned above. Key respondents were considered local experts or people in the study area with knowledge of a particular issue or technology of interest (in this case, traditional animal healthcare knowledge) [12-14]. They have a more extensive understanding of local social and veterinary-cultural systems than others in the community. A purposive sample referred to a particular subset of knowledgeable people in the area of traditional animal healthcare system. Intensive and extensive collaboration and interaction with these key respondents was considered an effective research strategy of accessing the relevant information [15,16]. A probability random sampling technique would not have been appropriate for this type of socio-cultural set-up, as not everyone sampled randomly may have the required knowledge [12,17-19]. A combination of snowball and purposive sampling methods was employed to select the key respondents. Once a few ethnopractitioners had been identified using the above sources, fruitful initial contacts were made and more ethnopractitioners were identified using their existing networks. Upon the establishment of the snowball sample, a purposive sampling technique was then employed to select a sample of 21 key respondents from Buuri district. This procedure is widely used in ethnoknowledge studies to get information from hidden populations, which are difficult for researchers to access [7,20-23]. The purposive sampling technique ensured that only key respondents with the desired qualities and quantities of information on traditional animal healthcare system of the Meru people were selected [24].

### Administration of questionnaire to key respondents

Each of the 21 key respondents was asked to fill a well structured questionnaire with the help of the interviewer. The questionnaire consisted of 18 questions requiring: - (1), the location where questionnaire is administered (village), (2), identity of the person being interviewed (name, sex, age, level of education, occupation etc.), (3), respondent’s consent agreement, (4), type of ethnoveterinary medicine practiced and how it was acquired, (5), the type of animals treated, (6), how the remedial products are identified, prepared, stored and administered, (7), how animals are treated and monitored, (8), how ethnopractitioners are paid for the services, (9), how ethnoveterinary medicine knowledge is shared amongst ethnopractitioners, (10), livestock diseases and/or ill health conditions treated, (11), plant and/or plant products used and their state/form, (12), state of affairs of the plant and/or plant products used for treatment, (13), factors contributing to the state of affairs of the plant and/or plant products used for treatment, (14), measures being taken for the state of affairs of the plant and/or plant products used for treatment, (15), challenges facing the profession of ethnoveterinary medicine, (16), personal opinion of the interviewee regarding the profession of ethnoveterinary medicine, (17), what should be done to improve traditional veterinary services in the interviewee’s area, and (18), personal observation of indications of practicing ethnoveterinary medicine made by the interviewer in the homestead of the interviewee.

Each time a questionnaire was administered to the interviewee, a senior relative/friend and a representative of the local administration from the office of the area sub-chief who was familiar with interviewee, were requested to accompany the interviewer. These two people engaged the interviewee into an interactive and productive discussion as the questionnaire was filled by the interviewer. This composition formed a very productive interaction that provided an enabling environment for Rapid Rural Appraisal (RRA) and Participatory Rural Appraisal (PRA) research to take place successfully. This method was considered very useful and robust because it reduced the following sources of bias: - (1) modelling bias, which was the projection of the interviewer’s views on to those studied, (2) strategic bias, which was the expectation of benefits by the subject, (3) familiar relationships between interviewer and interviewee (senior relative, administrator representative and interviewee) which would reduce resistance to questioning but could lead to rote answers and outsider bias and (4), reduction of “key personae” bias [25]. These preconceived notions would therefore lead to incorrect filling of the questionnaire and poor documentation and analysis of the collected information [12,13].

### Personal interviews/discussions with selected key respondents

After filling of the well structured questionnaire, an interview/discussion with the selected key respondents was held. This was guided exchanges, semi-structured by a mental checklist of relevant points to confirm the validity of the information in the questionnaires of other key respondents interviewed earlier.

### Collection of specimens of plants and plant products

Following a personal interview with the selected key respondents, a field trip was made to identify and collect the listed plant specimens and/or ethnobotanical products. The specimens were harvested, prepared, packaged and stored according to the herbarium rules and regulations until transported to Herbarium at The Catholic University of Eastern Africa, Nairobi, Kenya for botanical identification using voucher specimens and according to the Hutchinson system of plant taxonomy based on the plants’ probable phylogeny. While in the herbarium, further non-experimental studies were also conducted. For each plant species collected from the field, a voucher specimen was prepared and deposited in the Herbarium at The Catholic University of Eastern Africa, Nairobi, Kenya.

### Collection of secondary data on traditional animal healthcare system

As part of non-experimental validation process of documented plants and plant products used in traditional animal healthcare system amongst the Ameru and evaluate their potential effectiveness, a systematic collection of secondary data on traditional animal healthcare system of the Meru people from the County Veterinary Office (CVO) preceded. This was followed by an extensive literature search on the taxonomy of the plant specimens collected and their ethnobotanical applications from the internet, livestock research institutions, non-governmental organizations (NGOs) and Herbarium libraries and laboratories. All these methods comprised a set of triangulation approach needed in ethnoveterinary medicine for the process of non-experimental validation [26].

### Enumeration of documented plants and plant products

A list of plants and plant products traditionally used to manage animal health amongst the Ameru, including their scientific and vernacular names, growth habits, family names, disease and ill-health conditions treated, target type of livestock and the preparation forms of different remedies was made (Table 1). The names of plants were arranged according to their alphabetical order. In Table 1, the classification of the plant specimens into growth life forms and/or habits was based on the definition and description of Yumoto et al. [27].

**Table 1:** Enumeration of documented plants and plant products traditionally used in health management of livestock by the Ameru of Buuri district, Meru County, Kenya (n = 48)

**Botanical name [Family]**	**Local name**	**Part(s) of plant used**	**Disease/ill-health condition treated and (the target type of livestock) [local name]**	**Method(s) of preparation**	**Herbarium voucher plant specimens’ number**	**References in literature supporting the claimed uses**
*Acacia drepanolobium* Harms ex sjostedt [Fabaceae]	*Muruai*	Bark	Retained placenta (c) [*Kuremera thigiri*]	Decoction	K/M/B/12-2011/015	[9,28,29]
*Acacia mearnsii* De Wild. [Fabaceae]	*Muthanduku*	Leaves	Coughing (c, g, s, p, pg, r) [*Gukolora*]	Infusion	K/M/B/12-2011/004	[30,31]
*Acacia xanthophloea* Benth. [Fabaceae]	*Murera*	Bark	Foot and Mouth disease (r, p, c) [*Ikunguri*]	Decoction	K/M/B/12-2011/029	-
*Ajuga remota* Benth. [Lamiaceae]	*Kirurite*	Leaves Whole plant	East Coast fever (c) [*Itaa/Ng’arang’ari*], Newcastle disease (p) [*Kuthinka*] and Helminthiasis (c, p, g, s, pg) [*Njoka*]	Infusion Cold infusion Decoction	K/M/B/12-2011/016	[29,30,32]
*Ajuga remota* Benth. [Lamiaceae]	*Kirurite*	leaves	Lung diseases (c, g, s, p) [*Mauri*]	Decoction	K/M/B/12-2011/016	-
*Allium cepa* L. [Liliaceae]	*Gitunguru*	Bulb	Bloat (c) [*Kuuna*]	Concoction	K/M/B/12-2011/034	[9,33-36]
*Aloe latifolia* Haw. [Aloaceae]	*Cukurui*	Leaves	Helminthiasis (c, p, g, s, pg) [*Njoka*], Diarrhoea/dysentery (c, p, g, s, pg) [*Kuarwa*] and Sores/Chronic wounds (c, g, s, p, pg ) [*Ironda*]	Decoction Decoction Leaf gel	K/M/B/12-2011/036	[7,37,38]
*Azadirachta indica* A. Juss [Meliaceae]	*Mwarubaine*	Leaves Roots	Helminthiasis (c, p, g, s, pg) [*Njoka*] and General weakness/dullness (c, g, s, p, pg) [*Kuaga inya*]	Decoction Decoction	K/M/B/12-2011/006	[7,9,33,35,36,39-41]
*Cannabis sativa* L. [Cannabaceae]	*Bangi*	Leaves	Diarrhoea/dysentery (c, p, g, s, pg) [*Kuarwa*], Pneumonia (p) [*Mpio*], and Newcastle (p) [*Kuthinka*]	Cold Infusion	K/M/B/12-2011/037	[42-44]
*Capsicum annuum* L. [Solanaceae]	*Nchini*	Fruits	Diarrhoea/dysentery (c, p, g, s, pg) [*Kuarwa*] and dullness (p) [*Kuaga inya*] Newcastle disease (p) [*Kuthinka*] and General weakness/dullness (c, g, s, p, pg) [*Kuaga inya*]	Decoction	K/M/B/12-2011/022	[7,9,33,38,41,43,45]
*Capsicum frutescens* L. [Solanaceae]	*Nchini*	Fruits	Diarrhoea/dysentery (c, p, g, s, pg) [*Kuarwa*] and General weakness/dullness (c, g, s, p, pg) [*Kuaga inya*]	Decoction	K/M/B/12-2011/038	[7,33]
*Carissa spinarum* L [Apocynaceae]	*Mukawa*	Roots Roots	Infertility (s, g) [*Kuthata*] Poor milk let down (c) [*Kuitha iria*] Mastitis (c) [*Kuimba riere*] and Miscarriage (c) [*Guta Njau*]	Decoction	K/M/B/12-2011/043	[9,34,46]
*Chrysanthemum cinerariaefolium* Vis. [Asteraceae]	*Mbeniko*	Flowers	General ectoparasites (c, p) [*Ngumba*] Specifically Mites, lice and fleas infestation (c, p) [*Nthuuga*] and Ticks’ infestation (as an aetiologic agent) (c, p, g, s, pg) [Igumba*]	Concoction	K/M/B/12-2011/002	[7,47-51]
*Commiphora eminii* (Engl.) J.B. Gillett [Burseraceae]	*Mutunguu*	Leaves Bark	Coughing (c, g, s, p, pg, r) [*Gukora*], Anaplasmosis (c, r) [Ntigana*], Helminthiasis (c, p, g, s, pg) [*Njoka*] and Diarrhoea/dysentery (c, p, g, s, pg) [*Kuarwa*]	Infusion Decoction	K/M/B/12-2011/035	[39]
*Cordia africana* Lam [Boraginaceae]	*Muringa*	Leaves Bark	Eyes diseases(c) [Meetho*] and General weakness (c, g, s, pg) [*Kuaga inya*]	Leaves are crushed to extract juice	K/M/B/12-2011/019	[38-41]
*Crotalaria laburnifolia* L. [Fabaceae]	*Mucugucugu*	Roots	Helminthiasis (c, p, g, s, pg) [*Njoka*]	Decoction	K/M/B/12-2011/021	-
*Croton megalocarpus* Hutch. [Euphorbiaceae]	*Mukinduri*	Bark	Diarrhoea/dysentery (c, p, g, s, pg) [*Kuarwa*]	Decoction	K/M/B/12-2011/009	[7,29,37,40]
*Cucumis aculeatus* Cogn. [Cucurbitaceae]	*Kamungu*	Fruits	Anaplasmosis (c, r) [Ntigana*] Helminthiasis (c, p, g, s, pg) [*Njoka*] and Loss of feathers (p) [*Guta mbui*]	Concoction Fruits are crushed to extract juice	K/M/B/12-2011/040	[9,30,52]
*Datura stramonium* L. [Solanaceae]	*Sikisiki*	Leaves	General ectoparasites (c, p) [*Ngumba*] Specifically Mites, lice and fleas infestation (c, p) [*Nthuuga*], Ticks’ infestation (aetiologic agent) (c, p, g, s, pg) [Igumba*] Newcastle (p) [*Kuthinka*] and Foot rot (c, g, s, r, pg) [*Maronda maguru*]	Concoction	K/M/B/12-2011/030	[9,40,41,53]
*Dodonaea angustifolia* L.f. [Sapindaceae]	*Murema ngigi*	Roots	East Coast fever (c) [*Itaa/Ng’arang’ari*]	Decoction	K/M/B/12-2011/024	[30,46,54]
*Dovyalis caffra* Warb. [Salicaceae]	*Kariaba*	Fruits	Coughing (c, g, s, p, pg, r) [*Gukora*]	Ripe fruits are crushed to extract the juice	K/M/B/12-2011/010	-
*Ehretia cymosa* Thonn. [Boraginaceae]	*Murembu*	Roots	Anaplasmosis (c, r) [Ntigana*] and Diarrhoea/dysentery (c, p, g, s, pg) [*Kuarwa*]	Decoction	K/M/B/12-2011/041	[39,55]
*Erythrina abyssinica* Lam. ex DC. [Fabaceae]	*Muuti*	Bark and roots	Anaplasmosis (c, r) [Ntigana*] and Helminthiasis (c, p, g, s, pg) [*Njoka*]	Decoction	K/M/B/12-2011/020	[7,39,42]
*Euclea divinorum* Hiern. [Ebenaceae]	*Mukirinyei*	Fruits Roots	Anaplasmosis (c, r) [Ntigana*] and Constipation (c, g, s) [Ntigana*]	Decoction Decoction	K/M/B/12-2011/044	[7,29,30,55]
*Euphorbia candelabrum* Kotschy [Euphorbiaceae]	*Kibubungi*	Bark/ Latex	East Coast fever (c) [*Itaa/Ng’arang’ari*]		K/M/B/12-2011/001	[7,9,29,30,40,52,55]
*Ficus thonningii* Bl. [Moraceae]	*Mugumo*	Bark	Diarrhoea/dysentery (c, p, g, s, pg) [*Kuarwa*]	Decoction	K/M/B/12-2011/039	[7,30,40,55]
*Kigelia africana* (Lam.) Benth. [Bignoniaceae]	*Murantina*	Bark	Helminthiasis (c, p, g, s, pg) [*Njoka*] and Dystochia (an abnormal or difficult childbirth or labour) (c) [*Kuremera njau*]	Decoction	K/M/B/12-2011/023	[9,32,34,37,39,55]
*Lantana camara L.* [Verbenaceae]	*Muchomoro*	Leaves	Pneumonia (c, s) [*Mpio*] and Coughing (c, g, s, p, pg, r) [*Gukora*]	Decoction	K/M/B/12-2011/042	[7,39,40,49-51]
*Nicotiana tabacum* L. [Solanaceae]	*Mbaki*	Leaves	General ectoparasites (c, p) [*Ngumba*] and Specifically Mites, lice and fleas infestation (c, p) [*Nthuuga*]	Concoction Fumigation	K/M/B/12-2011/025	[7,9,29,42,55]
*Olea europaea* L. [Oleaceae]	*Mutero*	Bark	Helminthiasis (c, p, g, s, pg) [*Njoka*]	Decoction	K/M/B/12-2011/032	[7,9,30,33,46]
*Plectranthus barbatus* Andr. [Lamiaceae]	*Kijara*	Roots Leaves	Pneumonia (c) [*Mpio*] and Fresh wounds (c, g, s, pg) [*Gutemwa*]	Decoction Leaves are crushed and juice squeezed.	K/M/B/12-2011/028	[7,30,32,37,52]
*Plumeria alba* L. [Apocynaceae]	*Mubono*	Leaves	Fresh wounds (c, g ,s) [*Gutemwa*]	Leaves are crushed and juice squeezed.	K/M/B/12-2011/045	[39]
*Prunus africana* (Hook.f.) Kalkman [Rosaceae]	*Mueria*	Bark	Coughing (c, g, s, p, pg, r) [*Gukora*]	Concoction (boiled and mixed with honey)	K/M/B/12-2011/048	[7,46,53,55]
*Ricinus communis* L. [Euphorbiaceae]	*Muariki*	Leaves and Seeds	East Coast fever [*Itaa/Ng’arang’ari*] and Bloat (c) [*Kuuna*]	Concoction. Seeds are crushed to extract oil	K/M/B/12-2011/026	[7,30,39,42,45]
*Rumex abyssinicus* Jacq. [Polygonaceae]	*Muraiguna*	Leaves	Eye diseases (Conjuctivitis (c, p, g, s, pg) [Meetho*]	Juice is squeezed into the eyes	K/M/B/12-2011/003	[39]
*Senna didymobotrya* (Fresen.) Irwin & Barneby [Fabaceae]	*Kirao*	Leaves Roots	Anaplasmosis (c) [Ntigana*] and Helminthiasis (c, p, g, s, pg) [*Njoka*]	Decoction Decoction	K/M/B/12-2011/005	[7,30,35,39,52,55]
*Senna septemtrionalis* (Viv.) H. Irwin & Barneby [Fabaceae]	*Kirao*	Roots	Anaplasmosis (c) [Ntigana*] and Helminthiasis (c, p, g, s, pg) [*Njoka*]	Infusion	K/M/B/12-2011/011	[35,39,56]
*Solanecio mannii* (Hook.f.) C. Jeffrey [Asteraceae]	*Mutoromboro*	Leaves	Anaplasmosis (c) [Ntigana*] and Typanosomiasis (c, g) [*Mutombo*]	Decoction	K/M/B/12-2011/017	[7,30,55]
*Solanum indicum* L. [Solanaceae]	*Ntongu*	Fruits	Skin rashes (p) [*Weere*] and Loss of feather (p) [*Guta mbui*]		K/M/B/12-2011/046	[57,58]
*Solanum incanum* L. [Solanaceae]	*Mutongu*	Roots Fruits	Helminthiasis (c, p, g, s, pg) [*Njoka*], Lumpy Skin Disease (c) [*Ngoci*] and Foot rot(c, g, s, r, pg) [*Maronda maguru*]	Decoction Fruit extract Concoction	K/M/B/12-2011/031	[9,29,33,40,59]
*Stephania abyssinica* (Dill. & A. Rich.) Walp. [Menispermaceae]	*Gatamba nathi*	Roots	Diarrhoea/dysentery (c, p, g, s, pg) [*Kuarwa*]	Decoction	K/M/B/12-2011/007	[55]
*Synadenium compactum* N. E. Br. [Euphorbiaceae]	*Muthuuri*	Latex/ Stem	East Coast fever (c) [*Itaa/Ng’arang’ari*]	Latex is applied to the swollen lymph nodes.	K/M/B/12-2011/014	[6,30]
*Tagetes minuta* L. [Asteraceae]	*Mubangi*	Leaves	General ectoparasites (c, p) [*Ngumba*]	Concoction Fumigation	K/M/B/12-2011/027	[7,9,30,35,40,49,50,60]
*Tephrosia vogelii* Hook. f. [Fabaceae]	*Mucugucugu*	Leaves	General ectoparasites (c, p) [*Ngumba*]	Decoction	K/M/B/12-2011/047	[7,9,33,34,40,49,50]
*Tetradenia riparia* (Hochst.) Codd. [Lamiaceae]	*Kiaraka*	Leaves	Anaplasmosis (c, r) [Ntigana*], Typanosomiasis (c, g) [*Mutombo*] and Miscarriage (c) [*Guta Njau*]	Concoction / Decoction	K/M/B/12-2011/012	[6,39,42,44]
*Tithonia diversifolia* (Hemsl.) A. Gray [Asteraceae]	*Kingana*	Leaves	General ectoparasites (c) [*Ngumba*] and Helminthiasis (c, p, g, s, pg) [*Njoka*]	Concoction Decoction	K/M/B/12-2011/018	[7,30]
*Vangueria infausta* Burch. [Rubiaceae]	*Mubiru*	Roots	Pneumonia (c, g, s, p) [*Mpio*]	Infusion	K/M/B/12-2011/013	-
*Warburgia ugandensis* Sprague [Canellaceae]	*Muthiga/ Musunui*	Bark Leaves Roots	Helminthiasis (c, p, g, s, pg) [*Njoka*] Pneumonia (c, g, s, p) [*Mpio*] and General weakness/dullness (c, g, s, p, pg) [*Kuaga inya*]	Infusion Decoction	K/M/B/12-2011/033	[29,30,61]
*Zea mays* L. [Poaceae]	*Mpempe*	Maize cob	Retained placenta (c) [*Kuremera thigiri*]	Maize cob is burnt, grinded into fine powder and mixed with water	K/M/B/12-2011/008	[35,45]

**Key:*c***–cattle; ***g*–**goats; ***s*–**sheep; ***p***–poultry; ***pg*–**pigs; ***r***-ruminants; ***m***-mammals; Vernacular names of plants and animals’ disease(s) and/or ill-health condition(s) based on the consensus of key respondents and local government veterinarians working in Buuri district, Meru County.

*Name used to describe two or more closely related livestock diseases/conditions/ill-health.

### Authenticity of collected information and plant family use value

In order to evaluate the reliability of the information gathered, each key respondent was visited at least twice on the same idea to prove the validity of the information given out during the first visit before its final documentation. Information that significantly deviated from the original data collected during the first interview without support from the existing literature was either rejected or verified with other key respondents before being considered for use [46].

The family use value (FUV), which deals with the relationship between the total number of plant species within a given family and the sum use values for all the species identified from the field was calculated according to Hoffman and Gallaher [62] as follows:

(1) $$FUV=\sum UV_{s}/\left(n_{s}\right)$$

Where: -

UV_s_ = Use values for all the species within a given family.

n_s_ = Total number of species within a given family.

The family use value is an important Relative Cultural Importance (RCI) index, which can be applied in ethnobotany to calculate a value of biological plant taxon. This index together with other important ethnobotanical indices can provide data that can be used in hypothesis-testing, statistical validation and comparative analysis [62].

### Respondent consensus factor

To estimate the variability of documented knowledge of ethnoveterinary medicine and determine the homogeneity of the information given by the key respondents, Respondent Consensus Factor (F_rc_) for the most common livestock diseases and/or ill-health conditions for the category of animal species with the number of reported remedial plants and/or plant products and/or ethnoformulations, were calculated based on Heinrich et al*.*[63] as follows:

(2) $$F_{\mathrm{rc}}=\left(n_{\mathrm{ur}}-n_{t}/n_{\mathrm{ur}}-1\right)$$

Where: -

n_ur_ = Number of usage-reports.

n_t_ = Number of taxa used.

In addition to defining how homogeneous the documented information is in the study population based on the degree of consensus in respondents’ responses, the F_rc_ values revealed the strength of reliance of respondents on various plants and plant products for the treatment of different livestock diseases and/or ill-health conditions [64]. The F_rc_ values range from 0 to 1. A high value (close to 1) indicated that there was a well-defined selection principle for certain specific plants and plant products traditionally used to treat livestock diseases and/or ill-health conditions in the community and/or there is sharing of information amongst the ethnopractitioners offering ethnoveterinary services in that particular community. A low value (close to 0) on the other hand indicated that plants and plant products used for the treatment of livestock diseases and/or ill-health conditions are chosen from a wide range of plants and plant products without relying on specific proven ones and/or the ethnopractitioners offering ethnoveterinary services do not share information amongst themselves.

## Results and discussion

### Respondents and their perception of knowledge of ethnoveterinary medicine

The selected 21 key respondents comprised mainly practicing ethnoveterinarians. The majority of respondents were males aged between 50–69 years old, with informal education (Table 2). Ethnoveterinary medicine knowledge was transmitted orally and secretly. The knowledge was maintained within family lineages and its services mostly offered freely (Table 2). By recognizing and involving ethnopractitioners in ethnoveterinary research and development in the community, they gradually started regaining confidence in their own EVM knowledge, services and practices, which had been previously condemned by the colonial governments and missionaries as witchcraft and satanic in nature. However, this state of affairs continued to its current condition because of the continued condemnation of EVM knowledge by the church and the failure of the succeeding African governments to legally recognize ethnoveterinary medicine knowledge and protect ethnopractitioners [7]. These findings are in agreement with numerous other studies previously carried out in other communities [7,65-71]. Most of these studies have revealed that the family as a unit is still a major source of ethnoknowledge for healing, training and gaining experience for many medical ethnopractitioners, whether for humans or animals. None of the respondents however attributed his/her ethnoveterinary medicine knowledge to have been acquired through personal experiences such as observations, experimentation, dreams and/or visions, an indication that there was probably good mentoring and/or apprenticeship, which ensured successful transmission of the desired knowledge through generations [6].

**Table 2:** A description of the profiles of key respondents and their perception of the acquisition, services and practices of ethnoveterinary medicine (n=21)

**S/n**	**Description of the categories of key informants**	**No. of respondents**	**Percentage (%)**
**1.**	**Gender**		
**a**	Male	18	86
**b**	Female	3	14
**2.**	**Age (yrs)**		
**a**	50 - 59	6	29
**b**	60 - 69	12	57
**c**	70 - 79	3	14
**3.**	**Education status**		
**a**	Formal education	5	24
**b**	Informal education	16	76
**4.**	**Acquisition of EVM knowledge and experience**		
**a**	From parents/grandparents/extended and non-extended family members	15	71
**b**	From an experienced senior ethnopractitioner not related	6	29
**c**	From own experience-dreams/visions	-	-
**d**	Ceremonies/meetings	-	-
**5.**	**Provision of EVM services**		
**a**	Not charging (free)	9	43
**b**	Always charging	5	24
**c**	Charging under certain circumstances only	7	33
**6.**	**Exchange of EVM knowledge amongst professionally experienced colleagues**		
**a**	Yes	4	19
**b**	No	17	81
**7.**	**State of EVM knowledge/services/practices**		
**a**	Falling in disfavour	5	23.8
**b**	Gaining ground	10	47.6
**c**	Status quo	6	28.6

**Key:** EVM – Ethnoveterinary medicine.

**N/B:** The ages of key respondents were confirmed from their: - (1), birth certificates and (2), national Identity Cards (ID)/passports in Kenya.

### Naming of plants amongst the Ameru

The survey of plants and plant products amongst the Ameru showed that they had a well defined system of naming both indigenous and foreign plants in their community (Table 1). Plant ethnosystematic amongst the Ameru is based on a number of factors, more particularly on the characteristics of the plants. For example, *Murema ngigi* (*Dodonaea viscosa* Jacq var. *angustifolia* (L.f) Benth.) and *Kirurite (Ajuga remota* Benth*)* are local *Kimîîru* names given to the two plants in reference to their hardness and bitter taste, respectively. In addition, the phenomenon of giving a single name to a large group of plants such as a family because the appearance of the plants is the same is very common amongst the Ameru but very challenging to modern taxonomists studying ethnobotany of such communities. This can render the process of correct identification of individual plant species within such a large group very perplexing. For example, most of the tree species in the *Acacia* genus are given one collective local *Kimîîru* name, *Miruai* (singular - *Muruai*). Similarly, *Muthuri* (plural - *Mithuri*) is a collective local *Kimîîru* name given to a large group of plants especially those that produce latex whether they belong to the same family/genus/species or not. This type of naming plants pose great dangers of erroneously using a given plant and plant products to treat a given disease and/or ill-health condition [6]. From the foregoing, it is self-evident that the *Kimîîru* dialects have both singular and plural forms of naming plants. For instance, a name of the plant species especially the trees starting with the prefix, *Mu-* normally signifies the singular form while the prefix *Mi-* represents the plural form (Table 1) [39]. For example, *Acacia mearnsii* De Wild. is known as *Muthanduku* in singular form and *Mithanduku* in plural form. This is the same case for *Cordia africana* Lam., which is locally known as *Muringa* and *Miringa* in singular and plural forms, respectively.

Because of the ethnic diversity amongst the Ameru living in the study area (Buuri district of Meru County), more than one vernacular name could be used to refer to the same plant species by different sub-tribes and vice versa (Table 1). The ethnic diversity affected a great deal new plant species brought in the community as they could be found with more than one *Kimîîru* name such as the case of *Warburgia ugandensis* Sprague. Plant species that were not indigenous to the Meru region had been given local names, which are descriptive in nature or took the altered form of the name used in their original language. An example is *Azadirachta indica* A. Juss whose local name *Mwarubaine* was apparently derived from the *Swahili* name, *Muarubaini*, which means ‘the tree of the fourty’, as it is believed to be able to treat more than fourty different diseases. Another example is *Cannabis sativa* L. whose local name, *Bangi* is similarly derived from the *Swahili* name for the plant. According to some key respondents, a local *Kimîîru* name could be used to refer to different plant species by different sub-tribes. For example, among the Imenti sub-tribe, *Ajuga remota* Benth is known as *kirurite* whereas the Tharaka sub-tribe use the same name to refer to *Tithonia diversifoli* (Hemsl.) A. Gray. Most key respondents however, were much aware of such divergence in naming local plants among different sub-tribes and were quick to point them out for discussion and building consensus (Table 1).

### Enumeration of documented plants from the survey study

A total of 48 plant species distributed in 26 families were documented to be used in livestock health management by traditional animal healthcare providers in the study area (Table 1). An extensive literature search was undertaken to evaluate literature that supports the claimed uses of the documented plants species (Table 1). Some of the documented plant species were reported in literature to be used in ethnomedicine and other cultural activities of other communities. Some plant species had very few ethnoknowledge references in the literature (only 1 or 2 references in literature) while the rest did not have any reference in literature such as *Acacia xanthophloea* Benth., *Crotalaria laburnifolia* L., *Dovyalis caffra* Warb and *Vangueria infausta* Burch; perhaps, they were truly indigenous to the Meru people or perhaps relevant references could not be accessed in literature. Those plants thought to be indigenous to the Meru community and traditionally claimed to manage animal health, were more than 14. Of the documented 48 plant species, some, such as *W. ugandensis, Tagetes minuta* L. and *A. indica* were already confirmed medicinal plants that had been studied for their use in ethnoveterinary medicine [7,9,28,30,33,40].

### Growth life forms of the documented plant species

**Figure 2:**
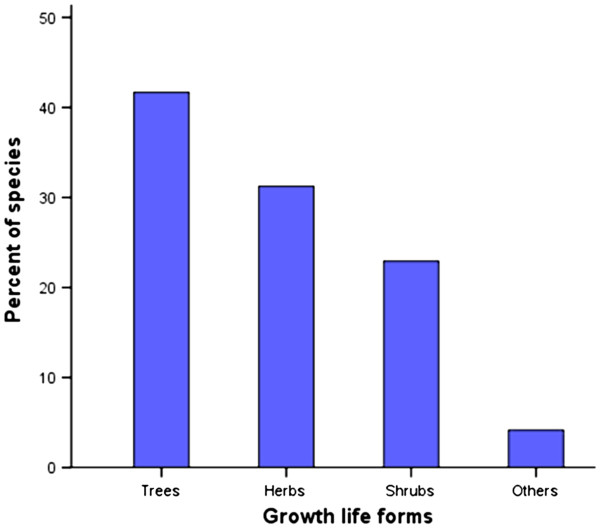
The growth life forms of documented plant species used in livestock health management in Buuri district, Meru County, Kenya (n=48).

Growth life forms of the documented plant species was categorized according to the description of Yumoto *et al*. [27]. An analysis of the growth life forms/habits of plants used by traditional animal healthcare providers in livestock health management in Buuri district revealed that trees constitute the largest category (41.7%), followed by the herbs (31.2%). Shrubs constituted 22.9% of the total recorded plant species while the rest, which included climbers and lianas constituted 4.2% (Figure 2). This shows that the most widely used plant habit in the study area is tree and this may be attributed to a number of factors among them the high level of abundance of trees in the area and hence easily accessed [39].

### Parts of plants used and preparation methods

**Figure 3:**
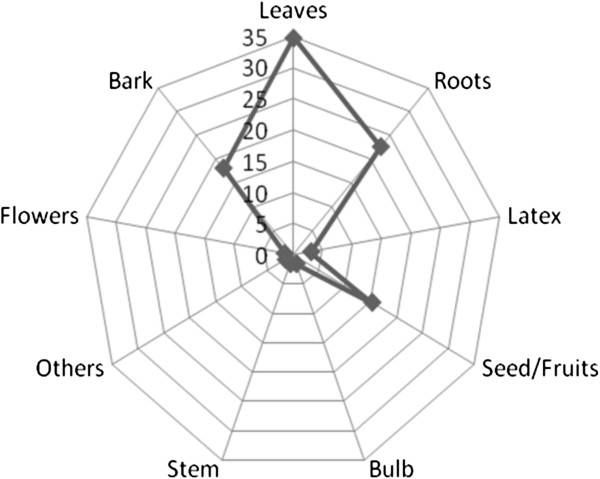
Percent distribution of plant parts used in ethnoveterinary medicine in Buuri district, Meru County, Kenya.

**Figure 4:**
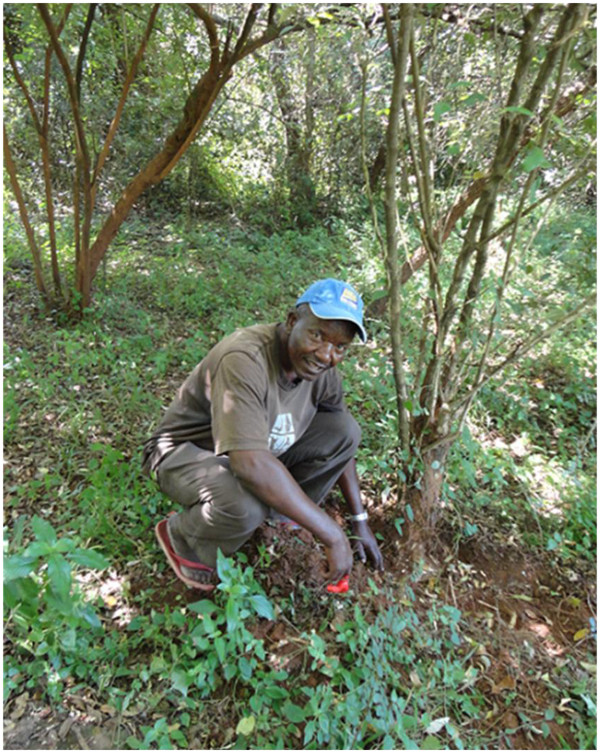
A key respondent demonstrating the process of harvesting roots from a medicinal plant in the forest in Buuri district, Meru County, Kenya.

In regard to the part(s) of plants harvested and used in ethnoveterinary medicine in Buuri district, the study revealed that the most frequently utilized part of the plant was the leaf accounting for 34.8% of the total reported ethnoformulation preparations followed by the root (22.7%), bark (18.2%), seed/fruit (15.2%), latex (3%) and bulb, flower and stem each accounted for 1.5% of the total reported ethnoformulation preparations in that order (Figure 3). These results are in an agreement with the previous findings of Amri and Kisangau [72], who conducted a similar survey study in villages surrounding Kimboza forest reserve in Tanzania but were contrary to the findings of Rukia [73]. Leaves from plants therefore appear to be the most preferred harvested parts of plants by ethnopractitioners for us in ethnomedicines [6]. Putting into consideration the biological function of the leaves on plants, the method of harvesting medicinal plants by picking leaves can be very devastating and a threat to the survival of the target plant, more particularly, if the young tender leaves are harvested instead of the old ones, which are almost dropping off the plant to become humus. Similarly, frequent harvesting of roots and barks, the second most preferred parts of plants (Figure 4), may be destructive and unsustainable, thus risking the extinction of the target plant species, and is therefore not advisable [74,75].

Ethnoveterinarians in the study area employed a number of methods for preparing herbal remedies. These methods largely depended on the type of the plant used, parts of plants employed, type of disease/ill-health condition and the animal species being treated [7]. Some of the most frequently used methods of ethnoformulation preparations in the study area included: - decoction, infusion, concoction and fumigation (Table 1). The survey study further found out that most of the remedies were prepared from a single plant species. Other prominent preparations however, involved the mixtures of different plant species and at times addition of one or more non-plant ingredients or additives such as milk, soup, honey, porridge, animal fat, salt etc. The use of more than one plant to make ethnoformulations are commonly used in the study area and respondents believed that such an ethnoformulation conferred some synergistic effects to the herbal remedies in certain cases where ingredients of two or more plants were considered to be more effective against a particular disease/ill-health condition than the use of individual plants separately. For example, a number of key respondents interviewed cited the use of a concoction of *Tetradenia riparia* (Hochst.) Codd and *Cucumis aculeatus* Cogniaux as one of the most effective remedy against anaplasmosis in cattle against using only one plant species for preparation and application of the herbal remedy. On other hand, the use of more than one plant to make ethnoformulations was believed to neutralize toxicity effects and/or bitterness of one part of the ethnoformulation preparation to make it palatable and easily administered. While making the remedial preparations from plants and plant products, the most frequently used solvent was water, particularly during the preparations of decoctions, concoctions and infusions with the addition of the above mentioned additives (milk, honey, animal fat and salt). However, there were some contradictions in a few cases among some informants on the type of additives used in preparations of some herbal remedies. For example, while a number of informants mentioned milk as an important additive for some of the remedies, others held the view that generally, milk reduced the potency of most herbal remedies and should not be used as an additive. However, this point of view depends largely on one’s ethnicity and cultural belief and taboos [7].

Many key respondents revealed that they rarely stored their drugs for future use but rather are used as soon as they are prepared from fresh plant materials. According to the key respondents, this was based on the belief that most of the remedies derived from plants and plant products lose their efficacy and curative power once stored for a long period of time following harvesting and preparation and the underlying science for this belief just goes beyond this work to speculate on. According to some respondents however, a few parts of the plants, such as the bark of *W. ugandensis* and *Commiphora eminii* Engl. were normally preserved in the roof of houses for future use though not for a very long time.

### Type of livestock treated using ethnoveterinary medicines in the study area

**Figure 5:**
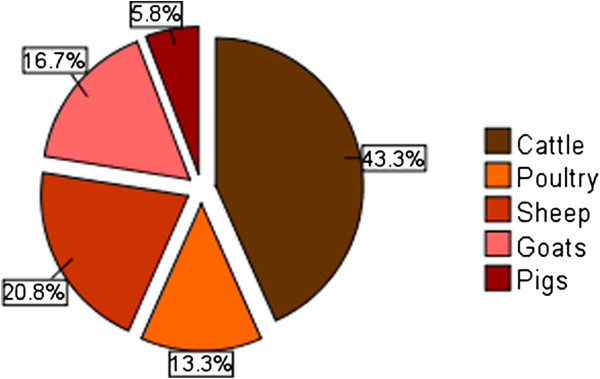
Ethnoremedies used on different animal groups in Buuri district, Meru County, Kenya.

The most commonly treated animals in Buuri district were: - cattle, goats, sheep, pigs and various species of poultry. Donkeys were also kept by few livestock farmers especially in the drier part of the district but no single plant and/or plant product was reported to be used by respondents in the treatment of donkeys. This was an indication that equine ethnoveterinary medicine might be less developed in the study area and/or perhaps the socio-economic value of donkeys in the cultural and traditional life of Ameru is not as great as the rest of the other animals. Majority of the livestock found in the study area were either indigenous breed or crosses between indigenous and exotic breeds. Cattle had the highest number of known ethnoveterinary remedies (43.3%) followed by sheep (20.8%), goats (16.7%) and poultry (13.3%) in that order. Pigs had the lowest number of recorded ethnoveterinary remedies (5.8%) (Figure 5). The number of known ethnoveterinary remedies for a particular type of livestock may probably correspond with socio-economic value and importance of the animal in the cultural and traditional life of Ameru [4,5] and perhaps this may also explain the order of acquisition of these animals for domestication by the Ameru in their life history. For instance, dowry among the Meru consisted of five items (an ewe, a container of honey, a heifer, a ram and a bull). All these items signified very important aspects of the marriage life with ewe symbolizing virginity. On the other hand, livestock (such as cattle, sheep, goats and donkeys) is believed to have been used by the leader of the Ameru people (*Koomenjoe*) to perform the second and the fourth tests of the five tests requested for by their colonial masters before the community could be released from bondage in a place called *Mbwa*[5].

### Ethnodiagnosis, determination of causes and naming of livestock diseases

Like in most African communities such as the Maasai [37], ethnodiagnosis of livestock diseases/ill-health conditions (Table 1) among the Meru people took a holistic view where the cause determined the type of the management strategy and/or treatment system to be adopted. Both human and livestock diseases/ill-health conditions were believed to have a multiplicity of causes [7,34]. Some livestock diseases/ill-health conditions were believed to be caused by pathogens and/or aetiologic agents that were ectoparasitological, endoparasitological and intraparasitological in nature while others were as a result of adverse weather conditions and were mostly seasonal. Some livestock diseases/ill-health conditions were believed to have a spiritual origin and such cases were dealt with spiritually through ritualism and exorcism by appealing to higher powers of spirits of the Ameru community. An accurate knowledge about the symptoms, signs and possible vectors of a particular disease was an important skill that preceded the choice of an appropriate treatment and management strategies. In making ethnodiagnoses, traditional animal healthcare providers based their conclusions on an in-depth understanding and comparative analysis of the general health versus ill-health signs [34]. Ethnodiagnosis was often carried out by the use of senses such as visual, audio, olfaction and tactile [34,37]. Depending on the nature of the disease/ill-health condition, ethnodiagnosis also involved consulting the spirits, oracles or divination and could at times involve the use of other animals [34]. Proper ethnodiagnosis of livestock diseases/ill-health conditions required a lot of experience and expertise and was greatly based on the knowledge of the diseases symptoms and signs, knowledge of known vectors, history of the environment and seasonality of disease outbreaks in addition to the knowledge of livestock species affected [37].

**Figure 6:**
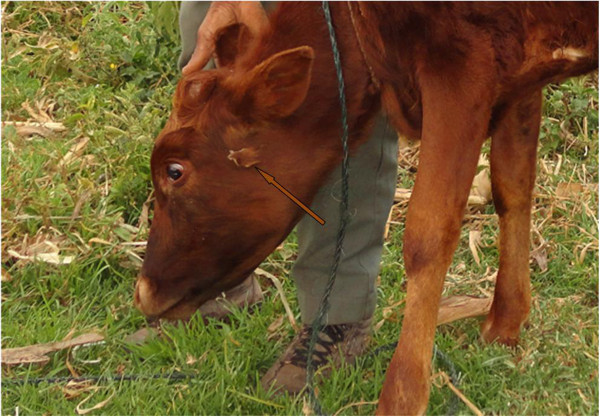
**A calf treated with the latex of *Synadenium compactum *N. E. Br. for East Coast fever (arrow show where the plant latex had been applied to the swollen parotid lymph gland).**

Naming of livestock diseases among the Ameru was not much different from that of other African communities. Just like in the naming of plants, some names for livestock diseases/ill-health conditions were descriptive in nature and related to aetiologic agents while other names did not have any relationship with the causative agents of the diseases/ill-health conditions, signs and/or symptoms (Table 1). About 30 livestock diseases and ill-health conditions were reported and described both in English and the local *Kimîîru* languages (Table 1). All the key respondents had at least one local name for the described diseases/ill-health conditions. The respondents were also able to describe various signs and symptoms associated with the reported diseases/ill-health conditions. Among the diseases/ill-health conditions described to have a high prevalence rate in the study area were: - anaplasmosis, East Coast fever (Figure 6), pneumonia and helminthiasis (Table 1). Most of the key respondents ranked EVM knowledge, services and practices as the most effective form of animal healthcare best suited for the majority of described diseases/ill-health conditions in comparison with the use of conventional medicine and services (Table 2).

### Administration methods and dosage of ethnomedicines used

**Figure 7:**
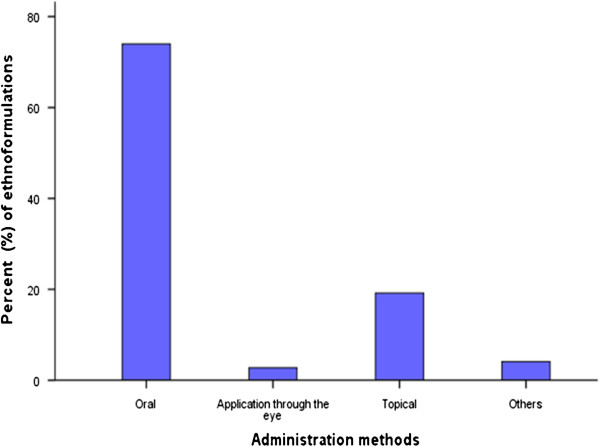
Routes of administration of ethnoformulations used by ethnoveterinarians in Buuri district, Meru County, Kenya.

The route of administration of ethnobotanical preparations depended on the nature of the disease and the target animal [7,34]. The main routes of administration documented in the study area were: - oral, topical/dermal, through the eyes and others such as application of the medicines directly on a fresh wound or cut. The most common route of administration was oral (74%) followed by dermal/topical (19.2%). Application of ethnomedicines through the eyes and other routes of administration accounted for 2.7% and 4.1%, respectively (Figure 7). Correct dosage (as described by an ethnopractitioner such as three glasses in a day) was an important aspect of ethnoveterinary medicine according to the respondents because, under dose was known to make the remedy ineffective while over dose caused livestock poisoning and subsequent death. Many respondents were of the opinion that the correct dosages for various ethnomedicines had been established through a lengthy period of trial and error mechanisms. Among the factors that determined the administration frequency and dose of the herbal remedies included: - the livestock species, age, body weight, level/state of illness and other conditions such as pregnancy and lactation. There were however, some discrepancies and difficulties in trying to determine the actual dosages for various ethnoformulation preparations from different respondents. This was largely due to the fact that measurements of most herbal remedies were administered through approximation and there existed little or no dosage standardization for most ethnoformulation preparations [34,59].

### The analysis of plant family use value

Of the 26 families, Fabaceae had the highest number of species (16.67%), followed by Solanaceae (12.5%), Asteraceae and Euphorbiaceae (each comprising 8.33%), Lamiaceae (6.25%), Apocynaceae and Boraginaceae (each comprising 4.17%), while the rest of the 19 families, each was represented by a single plant species (Tables 1 and 3). The plant family use value, which is applied in ethnobotany to calculate a value of biological plant taxon, helps in rating plant families for overall evaluation of member plant species in hypothesis-testing, statistical validation and comparative analysis [62]. From results presented in Table 3, Canellaceae, which was represented by a single plant species, was reported as the most useful family utilized in ethnoveterinary medicine in the study area followed by Apocynaceae, Boraginaceae, Lamiaceae, Aloaceae, Bignoniaceae, Euphorbiaceae, Moraceae and Polygonaceae in that order, some being represented by more than one single plant species.

**Table 3:** Analysis of documented plant species by family use values (n = 26)

**S/n family**	**No. of species**	**% of all species**	**Respondents’ use citations**	**% use citations**	**Family use value**
1 Aloaceae	1	2.1	12	2.45	0.571
2 Apocynaceae	2	4.2	29	5.92	0.690
3 Asteraceae	4	8.3	40	8.16	0.476
4 Bignoniaceae	1	2.1	12	2.45	0.571
5 Boraginaceae	2	4.2	29	5.92	0.690
6 Burseraceae	1	2.1	9	1.84	0.429
7 Canellaceae	1	2.1	19	3.88	0.905
8 Cannabaceae	1	2.1	5	1.02	0.238
9 Cucurbitaceae	1	2.1	9	1.84	0.429
10 Ebenaceae	1	2.1	8	1.63	0.381
11 Euphorbiaceae	4	8.3	48	9.80	0.571
12 Fabaceae	8	16.7	68	13.88	0.405
13 Lamiaceae	3	6.3	37	7.55	0.587
14 Liliaceae	1	2.1	9	1.84	0.429
15 Meliaceae	1	2.1	17	3.47	0.810
16 Menispermaceae	1	2.1	8	1.63	0.381
17 Moraceae	1	2.1	11	2.24	0.524
18 Oleaceae	1	2.1	10	2.04	0.476
19 Poaceae	1	2.1	9	1.84	0.429
20 Polygonaceae	1	2.1	11	2.24	0.524
21 Rosaceae	1	2.1	6	1.22	0.286
22 Rubiaceae	1	2.1	7	1.43	0.333
23 Salicaceae	1	2.1	6	1.22	0.286
24 Sapindaceae	1	2.1	10	2.04	0.476
25 Solanaceae	6	12.5	54	11.02	0.429
26 Verbenaceae	1	2.1	7	1.43	0.333

### Consensus building amongst key respondents on livestock diseases treated

**Figure 8:**
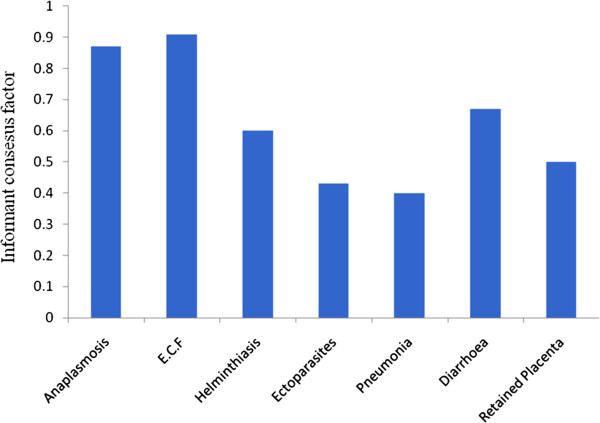
Respondent consensus factor for the main cattle diseases traditionally treated using plants and plant products in Buuri district, Meru County, Kenya.

Based on the reports from different respondents and looking at the numbers of ethnoformulation preparations described for each category of animal species, livestock farming may be one of the most important types of farming activities practiced by many farmers in the study area (Table 1). Cattle have a high socio-economic value and are a source of food, cash, manure, labour (ploughing and cart oxen) and as a means of dowry payment [4,5]. For this reason therefore, the interviewer sought to establish the key respondent consensus factor (F_rc_) for the main cattle diseases treated using different plants and plant products in Buuri district using formula (ii) and the results obtained are shown in Figure 8. The cattle disease that obtained the highest F_rc_ value was ECF (0.91) followed by anaplasmosis (0.87) and diarrhea (0.67) in that order. These are the most commonly encountered and perhaps well diagnosed diseases by traditional animal healthcare providers in the study area. The lowest F_rc_ value was obtained for pneumonia (0.4). Diseases with low F_rc_ values may be either new in the area or poorly diagnosed by the traditional animal healthcare providers. The F_rc_ defined how homogeneous the information was by the degree of consensus in key respondents’ responses.

## Conclusions

The survey revealed a wealth of preserved ethnoknowledge on plants, plant products and ethnopractices associated with the traditional management of livestock health by the Ameru. A total of 48 plant species distributed in 26 families were documented to be used in the management of livestock health by traditional animal healthcare providers. Of the 26 families, Fabaceae had the highest number of species (16.67%), followed by Solanaceae (12.5%), Asteraceae and Euphorbiaceae (each 8.33%), Lamiaceae (6.25%), Apocynaceae and Boraginaceae (each 4.17%), while the rest of the 19 families, each was represented by a single plant species. Majority of these 48 plant species were trees (41.7%) and herbs (31.2%). The most frequently utilized part of the plant was the leaf accounting for 34.8% of the total reported ethnoformulation preparations followed by the root (22.7%), bark (18.2%), seed/fruit (15.2%) and latex (3%) while bulb, flower and stem each accounted for 1.5% of the total reported ethnoformulation preparations. However, prominent ethnoformulation preparations (decoction, infusion, concoction and fumigation) involved the mixtures of different plant species and at times, the addition of one or more non-plant ingredients or additives such as milk, soup, honey, porridge, animal fat, salt etc. as this was believed to confer some synergistic effects to the herbal remedies and further make it easily administered. The most common route of administration of these ethnoformulation preparations was oral (74%) followed by dermal/topical (19.2%), through the eyes (2.7%) and other routes (4.1%) in that order. However, most herbal remedies were administered through approximation and there hardly existed dosage standardization for most ethnoformulation preparations.

Nevertheless, some of the local claims of the plants and plant products have been supported by scientific studies reported in literature. This therefore may imply that conducting in-depth scientific studies may help elucidate the science underlying the efficacy of these plants, plant products and health ethnopractices in managing animal health and this may lead to the discovery of useful pharmaceutical agents and tactics that may be integrated in livestock health management programmes for the wellbeing of livestock industry and human life in Africa. There is need therefore for the Ameru to address the challenges of sustainable utilization and conservation of these medicinal plants and plant products, more particularly educating all the stakeholders on sustainable methods of harvesting remedial products from plants and sustainable conservation mechanisms of creating woodlots in arable farming systems to relief constraints on the wild resource counterparts.

## Competing interests

The authors declare that they have no competing interests of any kinds and share the aspirations of the Meru people of central Kenya in their efforts to sustainably utilize and conserve their centuries’ old ethnoknowledge for future generations.

## Authors' contributions

WW designed data collection outline, analyzed data and equally contributed to the write up of the manuscript. MMG came up with the project idea, collected data and equally contributed to the write up of the manuscript. All the authors read and approved the final manuscript.

## Authors' information

WW is currently a research scientist and senior lecturer at the School of Pure and Applied Sciences, Department of Biological Sciences, South Eastern University College (a constituent college of the University of Nairobi) in Nairobi, Kenya. He holds a PhD in Resource Conservation and Production Ecology (in applied entomology, parasitology and ethnoknowledge) from Wageningen University and Research Centre, The Netherlands with a three-year laboratory and field research at the International Centre of Insect Physiology and Ecology (ICIPE) in Nairobi, Kenya. Currently, he is actively involved in ethnoknowledge, ethnomedicine and biomedical research in Kenya. MMG has just completed his Bachelor of Education degree in science at the Department of Natural Sciences, The Catholic University of Eastern Africa and is currently preparing to start his graduate studies in African Ethnobotany.
